# Determinants of successful smoking cessation in outpatient settings: A comparative analysis of varenicline and free program interventions

**DOI:** 10.18332/tid/208449

**Published:** 2025-09-24

**Authors:** Yaohong He, Jin Chen, Lihua Zhao, Shuang Qu

**Affiliations:** 1 Department of Respiratory, Fu Xing Hospital, Capital Medical University, Beijing, China

**Keywords:** smoking cessation outpatient clinic, smoking cessation, smoking nicotine dependence, analysis of influencing factors

## Abstract

**INTRODUCTION:**

Smoking cessation is a significant challenge, and various factors influence the success rates. Understanding the factors affecting cessation outcomes can guide more effective intervention strategies. This study compares the efficacy of a general outpatient cessation program versus a free cessation program, with subgroup analyses based on medication type (bupropion vs varenicline), aiming to identify factors associated with successful smoking cessation in outpatient settings.

**METHODS:**

We analyzed data from 356 patients visiting our smoking cessation clinic between January 2018 and June 2022, with random allocation into two groups via computerized random number table upon enrollment: Group A (general outpatient intervention, n=188) and Group B (free cessation program, n=168). Both groups were further subdivided based on the use of bupropion (A1, B1) or varenicline (A2, B2). Factors such as demographic data, nicotine dependence, smoking cessation confidence, exhaled carbon monoxide, and smoking cessation success (7-day point prevalence abstinence rate [PPAR] and 3-month continuous quitting rate [CQR]) were analyzed. To ensure comprehensive results, we performed an intention-to-treat (ITT) analysis, including participants who dropped out or did not complete the study as failures in the smoking cessation outcome.

**RESULTS:**

There were no significant differences between groups A and B in demographics, smoking behavior, or medication, except for occupation and duration of cigarette smoking. Group B had lower nicotine dependence but comparable smoking cessation outcomes to Group A. Factors associated with successful cessation included being older, married, employed full-time, smoking ≤20 cigarettes/day, and using varenicline. The CQR of Group A2 was 9.36% higher than that of Group A1. The PPAR and CQR of Group B2 were 16.66% and 17.93% higher than those of Group B1, respectively. However, there were no significant differences in PPAR and CQR between Group A2 and Group B2.

**CONCLUSIONS:**

Varenicline use, specific sociodemographic characteristics (aged >50 years, married, full-time employment), and less severe smoking behavior (≤20 cigarettes/day, ≤25 years duration, ≤600 pack-year) are key determinants of successful smoking cessation in outpatient settings. Varenicline use was associated with significantly higher cessation rates compared to bupropion within both intervention models. The free program incorporating varenicline (B2) demonstrated particularly high success rates.

## INTRODUCTION

Smoking is a health risk behavior, which can lead to oral cancer, lung cancer and other cancers. Nicotine, tar and other components of tobacco may also affect the fertility of men and women^1^. In addition, smoking during pregnancy is associated with pregnancy complications and adverse birth outcomes^2,3^. Mainstream smoke produced by smoking and sidestream smoke from smoldering tobacco products can also cause lung infections and wheezing in children in the form of secondhand smoke, increasing their risk of lymphoma, leukemia, liver cancer, or brain tumors^4,5^. Smoking is not only a major contributor to human mortality but is also preventable^6^; however, according to epidemiological statistics, the number of deaths due to smoking remains up to 8 million deaths every year, of which 7 million are caused by firsthand smoke, and as many as 1.2 million are related to secondhand smoke. At the same time, nearly 68.0% of adult smokers have the intention to quit smoking^7,8^.

Tobacco dependence is a major cause of global morbidity and mortality, with nicotine being the main psychoactive ingredient in tobacco^9^. Once used as an agricultural insecticide, nicotine is essentially a neurotoxin whose reward mechanism is regulated by neuronal nicotinic acetylcholine receptors to establish and maintain the body’s dependence on tobacco^10,11^. Smoking cessation can help cancer-diagnosed patients improve clinical efficacy and reduce the risk of death by up to 40%^12,13^. Tobacco dependence not only increases the burden on the healthcare system, but also poses a health threat to patients. Therefore, it is necessary to help smokers optimize their smoking cessation plans to reduce the burden on the healthcare system and promote the recovery of patient health.

Previous studies have shown that quitting smoking is not only beneficial for physical health, but also for improving mental health^14^. A systematic review and meta-analysis pointed out that smoking cessation reduced depression, anxiety, and stress while contributing to the maintenance of positive emotions and improvements in quality of life to some extent^15^. Based on the above, we believe that it is necessary to explore effective smoking cessation programs and the factors that affect smokers’ successful smoking cessation, which is not only conducive to further optimizing smoking cessation programs, but also has important clinical value for improving smokers’ related clinical outcomes. Many researchers have explored and analyzed smoking cessation in the past. For example, Matuszewski et al.^16^ indicated that the use of an exhaled CO monitor can greatly (71.0%) improve patients’ willingness to quit smoking and increase the chances of quitting smoking by 10 times, which is more conducive to the smooth promotion of quitting smoking in orthopedic trauma patients. In the study by Chen et al.^17^, the combination of exhaled CO measurement and self-declared smoking cessation in 33 outpatient smokers significantly improved smoking cessation, with the probability of successful smoking cessation within 3 months reaching 15.2%. The results of a clinic smoking cessation program showed that the smoking cessation program based on the clinical practice guideline of ‘Treating Tobacco Use and Dependence’ in combination with motivational interviewing was effective in five patients who smoked, with three patients achieving complete quitting and one reducing smoking behavior^18^.

There is currently limited research on factors influencing successful smoking cessation among smokers in smoking cessation outpatient clinics. This study mainly compares the influence of a free smoking cessation program and general outpatient interventions on smokers’ smoking cessation effects, and analyzes the influencing factors of successful smoking cessation among smokers in the smoking cessation outpatient clinic, in order to provide a theoretical basis for better implementation of free smoking cessation programs in the future.

## METHODS

### Study design

We analyzed data from 356 patients who visited the smoking cessation outpatient clinic of our hospital between January 2018 and June 2022. Ethical approval was obtained from the Ethics Committee of Capital Medical University (Approval number: 2024FXHEC-KSP028, on April 3, 2024), which specifically reviewed and approved the inclusion of minors aged 15–17 years. For participants aged 15–17 years, additional ethical safeguards were implemented, including mandatory written informed consent from both the minors themselves and their legal guardians (parents or legal custodians). The ethics committee ensured that the study procedures posed minimal risk to minors and that their rights to withdraw at any time were clearly communicated.

**Figure 1:**
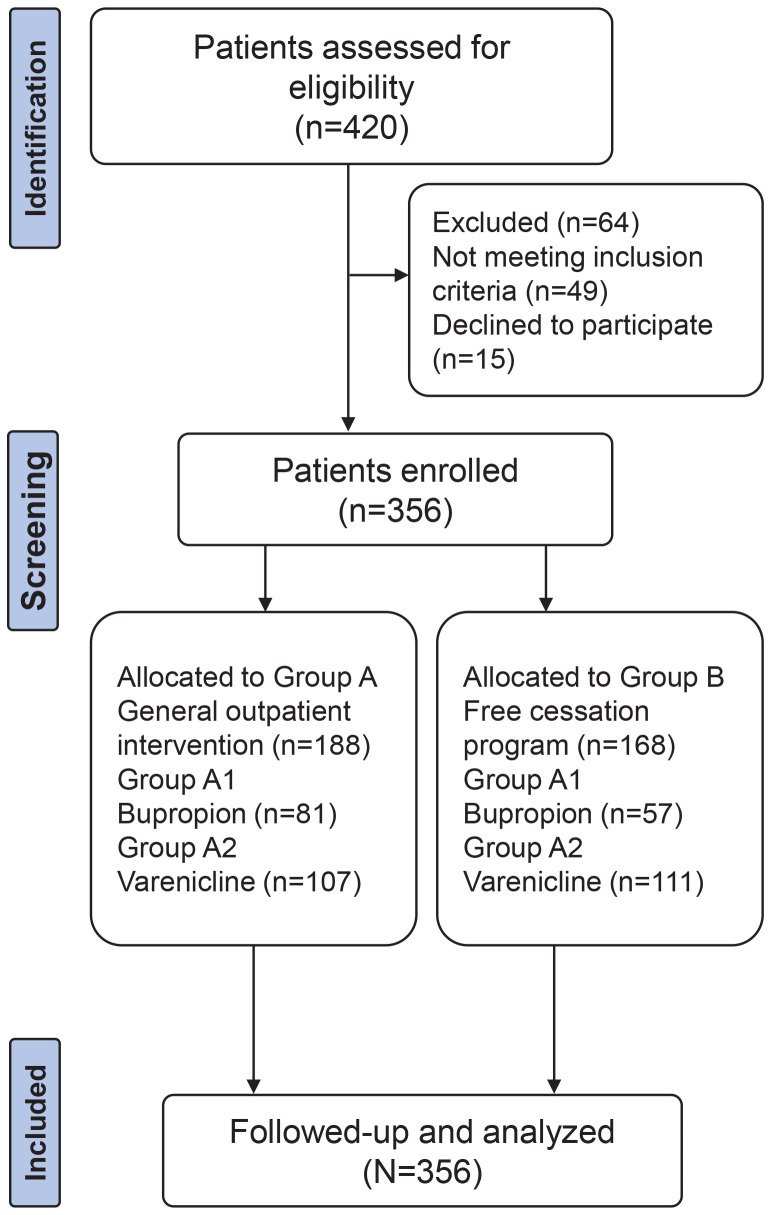
Flow diagram illustrating the process of patient selection, randomization, allocation, and analysis

After being approved by the Ethics Committee of our hospital and obtaining the informed consent signed by all the subjects, 356 patients admitted to the smoking cessation outpatient clinic of our hospital from January 2018 to June 2022 were selected as the research subjects. Then, 356 participants were randomly allocated into different intervention groups using a computerized random number table method upon enrollment. Among them, 188 cases in group A received smoking cessation interventions in the general outpatient clinic, and 168 cases in group B received smoking cessation interventions in a free smoking cessation program. The randomization process was conducted by a third-party statistician, who was blinded to the clinical characteristics of the participants. To ensure comparability between the groups, both were further subdivided based on the type of pharmacological intervention. Group A was subdivided into two subgroups: Group A1, which received bupropion, and Group A2, which received varenicline. Similarly, Group B was subdivided into Group B1, which received bupropion, and Group B2, which received varenicline. No significant differences were found between groups in terms of demographic data (p>0.05), suggesting clinical comparability. Moreover, to ensure comprehensive results, an ITT analysis was performed, including participants who dropped out or did not complete the study. In this analysis, all participants were treated as failures in the smoking cessation outcome, regardless of adherence or completion, thereby providing a more conservative estimate of the intervention’s effectiveness. The process of patient selection, randomization, allocation and analysis is illustrated in Figure 1.

### Inclusion and exclusion criteria

*Inclusion criteria*

Individuals were enrolled in the study if they met the following criteria as current and active smokers, defined as individuals who: 1) were aged ≥15 years; 2) smoked at least one cigarette daily for the past 30 days; 3) had a total lifetime cigarette consumption exceeding 100 units; 4) voluntarily agreed to participate in questionnaire surveys and telephone follow-ups; and 5) possessed complete clinical data. Adolescence (aged 15–17 years) is a critical period for smoking initiation and addiction development. Excluding this age group would limit the generalizability of findings to a high-risk population with unique cessation needs. Data from minors also provide insights into early intervention strategies, which are essential for reducing long-term smoking-related morbidity.

*Exclusion criteria*

Patients who did not meet the definition of current smoking with poor compliance (missing ≥3 follow-up visits or having a medication adherence rate <80%), cognitive dysfunction (assessed via the Mini-Mental State Examination [MMSE] with a score <24), psychiatric disorders (based on DSM-5 diagnostic criteria, confirmed by a psychiatrist), or mental illness, as well as those who were unwilling to cooperate in filling in the smoking cessation questionnaire, were excluded.

### Cessation methods

Group A received the following smoking cessation interventions in the general outpatient clinic: The subjects were registered for the first time with the ‘Smoking Cessation Outpatient Registration Form’ designed by the Tobacco Control Office at the Chinese Center for Disease Control and Prevention. Besides, a questionnaire survey was conducted, collecting information on demographic data, smoking status, and willingness to smoking cessation intentions. The investigation was conducted by specially trained smoking cessation doctors, and the intervention modalities (psychological intervention, behavioral intervention, smoking cessation medication, etc.) were determined according to the smokers’ addiction. The intervention period was at least one month. Participants were assessed for smoking cessation efficacy through face-to-face or telephone interviews.

Group B received smoking cessation interventions in a free smoking cessation program. First, the alliance uniformly trained the person in charge of the smoking cessation work of each member unit on smoking cessation methods. Smokers were then given interventions by the persons in charge of the smoking cessation program, including distributing brochures and conducting health education, covering smokingrelated diseases, benefits after quitting smoking, criteria for determining the severity of tobacco dependence, how to develop smoking cessation plans, common withdrawal reactions and coping methods, principles and usage methods of smoking cessation drugs, etc. A one-on-one lecture of 15–30 minutes was given to each smoker, and one course (3 months) of smoking cessation medication intervention was provided to smokers in need of smoking cessation assistance, especially those who were moderately or heavily dependent on tobacco.

We randomly assigned Groups A and B to Groups A1 and A2, and Groups B1 and B2, respectively. Groups A1 and B1 were given BUP hydrochloride sustained-release tablets orally: 150 mg was given once daily on days 1–3, 150 mg was administered twice daily on days 4–7 (with an 8-hour interval between the two doses), and 150 mg once daily was given after day 8 for a course of 8–12 weeks.

Groups A2 and B2 were treated with varenicline: 0.5 mg was administered orally on the first to third day, once a day; oral administration of 0.5 mg was given on the 4th to 7th day, twice a day; 1 mg was administered twice a day from the 8th day; the course of treatment was 12 weeks.

### Outcome measures

*Demographic data*

The demographic data of groups A and B were compared and analyzed, including gender, age, education level, marriage, occupation, monthly income, average monthly cigarette smoking cost, cigarettes smoked per day, duration of cigarette smoking, pack-year, and medication^19,20^.

*Nicotine dependence score*

The nicotine dependence was scored according to the internationally accepted nicotine dependence test scale, which contained 6 questions with a total score of 10 points. The degree of nicotine dependence is assessed on a cumulative score, with higher scores indicating more severe nicotine dependence in smokers^21,22^.

*Smoking cessation confidence*

Smokers were asked to self-rate themselves on a scale of 0–10, with higher scores indicating higher confidence in quitting^22,23^.

*Exhaled CO measurement*

The CO value in ppm was measured by an exhaled CO monitor^24^.

*Smoking cessation effect*

Seven-day point prevalence abstinence rate (PPAR): Assessed at 7 days post-intervention initiation, defined as self-reported abstinence from smoking for at least 7 consecutive days, confirmed by exhaled carbon monoxide (CO) measurement (CO ≤10 ppm). Three-month continuous quitting rate (CQR): Evaluated at 3 months post-intervention initiation, defined as self-reported continuous abstinence from smoking for the entire 3-month period, validated by both daily self-report diaries and exhaled CO measurement (CO ≤10 ppm at each follow-up visit)^25^.

### Statistical analysis

The data of this study were analyzed by Statistic Package for Social Science (SPSS) 23.0 (IBM, Armonk, NY, USA). Missing data were generated using multiple imputation to generate five imputed data sets, excluding cases with >30% missing data for key variables. Frequencies (n) and percentages (%) and mean ± SD were used to represent categorical and quantitative data, respectively. The χ^2^ test and Student’s t-test were used for comparisons of categorical and quantitative data, respectively, with the difference considered statistically significant at p<0.05.

## RESULTS

### Comparisons of baseline characteristics

The demographic data of the two groups (Group A and Group B) were compared, and no statistically significant differences were found in gender, age, education level, marital status, monthly income, average monthly cigarette smoking cost, number of cigarettes smoked per day, duration of smoking, pack-year, or medication use (all p>0.05; Table 1). Specifically, in terms of gender, Group A had 179 males (95.21%) and 9 females (4.79%), while Group B had 160 males (95.24%) and 8 females (4.76%). Regarding age, 45.21% of Group A and 54.76% of Group B were ≤50 years. Regarding education level, 48.94% in Group A and 54.17% in Group B had a college degree or below. Marital status was similar, with 94.15% in Group A and 97.62% in Group B married. In terms of occupation, 66.49% in Group A and 80.36% in Group B were full-time employees, and Group A had more retirees (26.60% vs 8.33%). For smoking duration, 60.11% in Group A and 49.94% in Group B smoked for >25 years. Pack-year was similar across groups (57.45% vs 64.88% for ≤600 pack-year). Medication use showed a slightly higher use of bupropion in Group A (43.09% vs 33.93%) and varenicline in Group B (66.07% vs 56.91%). Significant differences were found in occupation (p<0.001) and smoking duration (p<0.043), with Group B having a higher proportion of full-time employees and a shorter smoking duration. These differences suggest that smokers with full-time employment or shorter smoking durations may be more likely to participate in free smoking cessation programs.

**Table 1:** Comparison of baseline characteristics

*Characteristics*	*Group A (N=188)* *n (%)*	*Group B (N=168)* *n (%)*	*χ^2^/t*	p*
**Sex**			0.991	<0.001
Male	179 (95.21)	160 (95.24)		
Female	9 (4.79)	8 (4.76)		
**Age** (years)			3.236	0.072
≤50	85 (45.21)	92 (54.76)		
>50	103 (54.79)	76 (45.24)		
**Education level**			0.972	0.324
College degree or lower	92 (48.94)	91 (54.17)		
Bachelor’s degree or higher	96 (51.06)	77 (45.83)		
**Marital status**			2.647	0.104
Married	177 (94.15)	164 (97.62)		
Unmarried/divorced	11 (5.85)	4 (2.38)		
**Occupation**			20.701	<0.001
Employed full-time	125 (66.49)	135 (80.36)		
Retiree	50 (26.60)	14 (8.33)		
Other	13 (6.91)	19 (11.31)		
**Monthly income** (RMB)			0.228	0.892
<5000	76 (40.43)	69 (41.07)		
5000–7000	53 (28.19)	50 (29.76)		
>7000	59 (31.38)	49 (29.17)		
**Average monthly cigarette smoking cost** (RMB)			0.222	0.895
<400	71 (37.77)	67 (39.88)		
400–600	70 (37.23)	62 (36.90)		
>600	47 (25.00)	39 (23.21)		
**Cigarettes smoked per day**			0.012	0.914
≤20	103 (54.79)	93 (55.36)		
>20	85 (45.21)	75 (44.64)		
**Duration of cigarette smoking** (years)			3.644	0.043
≤25	75 (39.89)	85 (50.06)		
>25	113 (60.11)	83 (49.94)		
**Pack-year** (cigarettes/year)			2.060	0.151
≤600	108 (57.45)	109 (64.88)		
>600	80 (42.55)	59 (35.12)		
**Medication**			3.134	0.077
Bupropion	81 (43.09)	57 (33.93)		
Varenicline	107 (56.91)	111 (66.07)		

Group A and B indicate general outpatient clinic and free smoking cessation program, respectively.

* Based on χ^2^ or independent t-test, where appropriate. RMB: 1000 Chinese Renminbi about US$140.

### Nicotine dependence score

**Figure 2:**
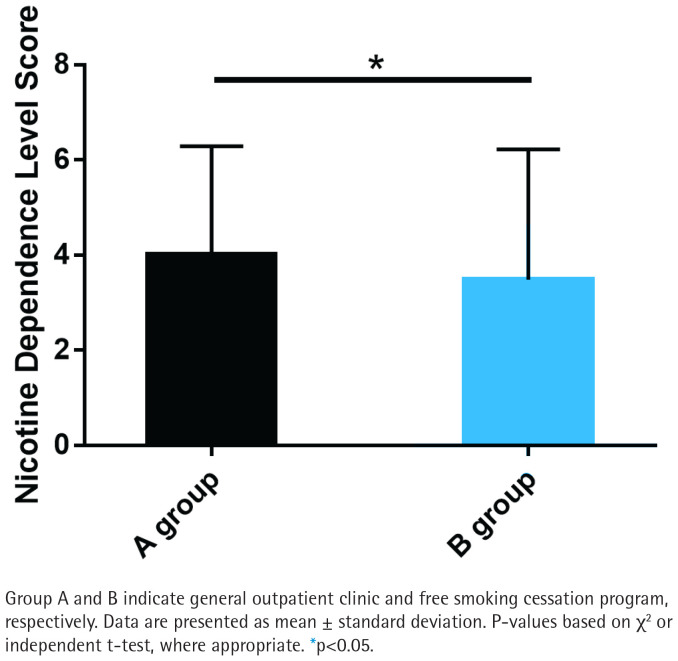
Nicotine dependence score between Group A and Group B

Group B exhibited significantly lower nicotine dependence scores compared to Group A (p<0.05; Figure 2). The mean nicotine dependence score in Group B was 2.8 ± 1.5, while in Group A it was 4.2 ± 1.8. This indicates that the free smoking cessation program may be more effective in reducing nicotine dependence than general outpatient interventions.

### Smoking cessation confidence

**Figure 3:**
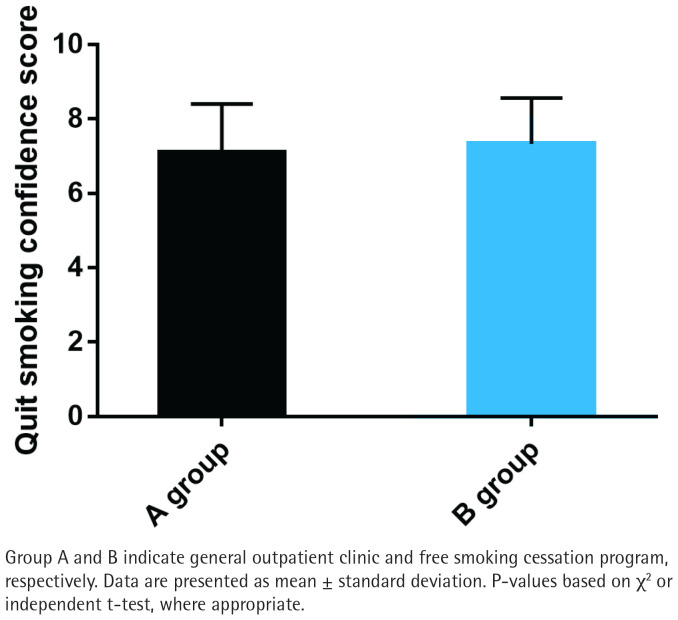
Smoking cessation confidence between Group A and Group B

While Group B reported slightly higher smoking cessation confidence scores (mean= 7.2 ± 1.1) compared to Group A (mean=6.8 ± 1.3), the difference was not statistically significant (p>0.05; Figure 3). This suggests that the free smoking cessation program does not significantly enhance smokers’ confidence in quitting, despite providing additional resources and support.

### Exhaled CO measurement

**Figure 4:**
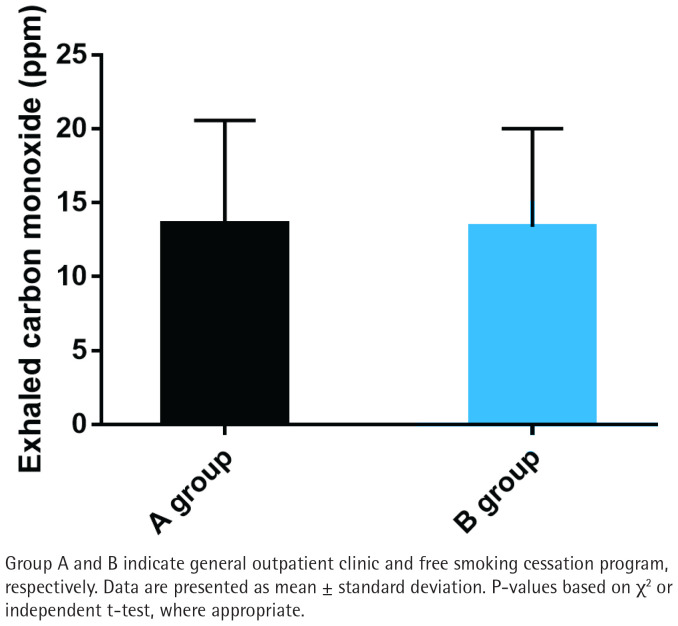
Exhaled carbon monoxide measurement between Group A and Group B

No significant differences were observed in exhaled carbon monoxide (CO) levels between the two groups (p>0.05; Figure 4). The mean exhaled CO level in Group A was 12.4 ± 4.7 ppm, while in Group B it was 11.8 ± 4.2 ppm. This implies that both interventions have comparable effects on reducing biological markers of smoking.

### Smoking cessation effect

Table 2 shows the effectiveness of smoking cessation interventions, evaluated using two primary outcome measures: PPAR and CQR. Group B (free smoking cessation program) demonstrated slightly higher PPAR (28.57% vs 27.66%) and CQR (41.67% vs 39.89%) compared to Group A (general outpatient intervention); however, these differences were not statistically significant (p=0.848 for PPAR and p=0.734 for CQR). This suggests that the free smoking cessation program and general outpatient interventions have comparable overall effectiveness in promoting short-term and medium-term smoking cessation. When stratified by medication type, significant differences in cessation effectiveness were observed. Group A2 (varenicline) achieved a significantly higher 3-month CQR (43.93%) compared to Group A1 (bupropion) (34.57%; p=0.002). Similarly, Group B2 (varenicline) exhibited significantly higher 7-day PPAR (34.23%) and 3-month CQR (47.75%) compared to Group B1 (bupropion) (17.54% and 29.82%, respectively; p=0.023 for PPAR and p=0.026 for CQR). Direct comparison between Group A2 (varenicline in general outpatient intervention) and Group B2 (varenicline in free program) revealed no significant differences in either PPAR (p=0.732) or CQR (p=0.571).

**Table 2:** Comparisons of smoking cessation effects between different clinics and medications groups

*Groups*	*Smoking cessation effects*	
	*Seven-day point* *prevalence* *abstinence rate* *n (%)*	*Three-month* *continuous* *quitting rate* *n (%)*
Group A (N=188)	52 (27.66)	75 (39.89)
Group B (N=168)	48 (28.57)	70 (41.67)
χ^2^	0.037	0.116
p	0.848	0.734
Group A1 (N=81)	13 (16.05)	28 (34.57)
Group A2 (N=107)	39 (36.45)	47 (43.93)
χ^2^	9.588	1.683
p	0.002	0.194
Group A1 (N=81)	13 (16.05)	28 (34.57)
Group B1 (N=57)	10 (17.54)	17 (29.82)
χ^2^	0.054	0.343
p	0.817	0.558
Group B1 (N=57)	10 (17.54)	17 (29.82)
Group B2 (N=111)	38 (34.23)	53 (47.75)
χ^2^	5.141	4.978
p	0.023	0.026
Group A2 (N=107)	39 (36.45)	47 (43.93)
Group B2 (N=111)	38 (34.23)	53 (47.75)
χ^2^	0.117	0.321
p	0.732	0.571

Group A1 and A2 indicate bupropion and varenicline in the general outpatient clinic, respectively. Group B1 and B2 indicate bupropion and varenicline in the free smoking cessation program, respectively. P-values based on χ^2^ test.

These findings suggest that varenicline-based interventions are more effective than bupropion-based interventions in achieving sustained smoking cessation, regardless of whether the intervention is delivered through general outpatient or free smoking cessation programs.

### Univariate analysis of successful 3-month continuous quitting

Univariate analysis was conducted to identify factors associated with successful 3-month continuous quitting. The results in Table 3 revealed significant associations between quitting success and several sociodemographic and smoking-related factors.

**Table 3:** Univariable analysis of successful 3-month continuous quitting

*Potential predictors*	*Quit success group* *(N=145)* *n (%)*	*Quit failure group* *(N=211)* *n (%)*	*χ^2^/t*	p*
**Gender**			2.188	0.139
Male	141 (97.24)	198 (93.84)		
Female	4 (2.76)	13 (6.16)		
**Age** (years)			5.537	0.019
≤50	62 (42.76)	94 (44.55)		
>50	83 (57.24)	117 (55.45)		
**Education level**			2.595	0.107
College degree or lower	82 (56.55)	101 (47.87)		
Bachelor’s degree or higher	63 (43.45)	110 (52.13)		
**Marital status**			4.364	0.037
Married	135 (93.10)	206 (97.63)		
Single/divorced	10 (6.90)	5 (2.37)		
**Occupation**			6.049	0.049
Employed full-time	116 (80.00)	144 (68.25)		
Retiree	19 (13.10)	45 (21.33)		
Other	10 (6.90)	22 (10.43)		
**Monthly income** (RMB)			4.310	0.116
<5000	68 (46.90)	77 (36.49)		
5000–7000	40 (27.59)	63 (29.86)		
>7000	37 (25.52)	71 (33.65)		
**Average monthly cigarette smoking cost** (RMB)			102.655	<0.001
<400	101 (69.66)	37 (17.54)		
400–600	34 (23.45)	98 (46.45)		
>600	10 (6.90)	76 (36.02)		
**Cigarettes smoked per day**			109.106	<0.001
≤20	128 (88.28)	68 (32.23)		
>20	17 (11.72)	143 (67.77)		
**Duration of cigarette smoking** (years)			18.494	<0.001
≤25	85 (58.62)	75 (35.55)		
>25	60 (41.38)	136 (64.45)		
**Pack-year** (cigarettes/year)			120.352	<0.001
≤600	138 (95.17)	79 (37.44)		
>600	7 (4.83)	132 (62.56)		
**Medication**			6.158	0.013
Bupropion	45 (31.03)	93 (44.08)		
Varenicline	100 (68.97)	118 (55.92)		
Nicotine dependence score, mean ± SD	1.99 ± 1.47	4.99 ± 2.35	13.64	<0.001
Confidence level, mean ± SD	8.02 ± 0.90	6.64 ± 1.18	11.87	<0.001

* Based on χ^2^ or independent t-test, where appropriate. RMB: 1000 Chinese Renminbi about US$140.

Participants aged >50 years had a higher rate of successfully quitting (57.24%) compared to those aged ≤50 years (42.76%). Married individuals had a higher quitting success rate (93.10%) compared to unmarried or divorced individuals (6.90%; p=0.037). Those employed full-time demonstrated a higher success rate (80.00%) compared to retirees (13.10%) or others (6.90%; p=0.007). Smokers with lower average monthly cigarette costs (<400 RMB) had a significantly higher quitting success rate (69.66%) compared to those with higher costs (17.54% for 400–600 RMB and 6.90% for >600 RMB; p<0.001). Daily cigarette consumption also played a role, with smokers consuming ≤20 cigarettes per day having a much higher success rate (88.28%) than those consuming >20 cigarettes per day (11.72%; p<0.001). Smokers with ≤25 years of smoking history had a higher success rate (58.62%) compared to those with >25 years (41.38%; p<0.001), and those with ≤600 pack-year had a significantly higher success rate (95.17%) compared to those with >600 pack-year (4.83%; p<0.001). Varenicline users showed a significantly higher quitting success rate (68.97%) compared to bupropion users (31.03%; p=0.013). Additionally, lower nicotine dependence scores (1.99 ± 1.47 vs 4.99 ± 2.36; p<0.001) and higher confidence levels (8.02 ± 0.90 vs 6.64 ± 1.18; p<0.001) were strongly associated with successful quitting.

These findings suggest that marital status, occupation, economic factors related to smoking, smoking intensity and history, medication type, nicotine dependence, and confidence level are all important determinants of successful smoking cessation in outpatient settings.

## DISCUSSION

In our analysis, groups A and B had similar data in terms of gender, age, education level, marriage, monthly income, average monthly cigarette smoking cost, number of cigarettes smoked per day, duration of cigarette smoking, pack-year, and medication, but there were significant occupational differences, indicating that smokers with full-time jobs are more inclined to choose free smoking cessation programs. Besides, statistically lower nicotine dependence scores were determined in group B compared with group A, suggesting that the free smoking cessation program received by group B is significantly better than the general outpatient intervention received in group A in reducing nicotine dependence. In terms of smoking cessation confidence, although the smoking cessation confidence score was slightly higher in group B compared to group A, there was no significant difference, indicating that the free smoking cessation program does not significantly increase smokers’ confidence in quitting. The exhaled CO data showed no significant difference between groups, indicating that the impact of free smoking cessation programs on exhaled CO is comparable to that of regular outpatient interventions. As far as the smoking cessation effect was concerned, the 7-day PPAR and the 3-month CQR were higher in group B than in group A, but with no significant difference, which indicates that the effect of the free smoking cessation program on smokers’ smoking cessation effect is similar to that of general outpatient interventions.

Further analysis of groups A and B based on different medication interventions revealed that the 3-month CQR in group A2 was significantly higher compared with group A1, while the 7-day PPAR and 3-month CQR in group B2 were significantly higher than those in group B1. This indicates that the intervention with varenicline has a more significant smoking cessation effect on smokers compared to bupropion therapy, mainly reflected in a higher 3-month CQR, and this effect is not affected by the intervention of general outpatient service or free smoking cessation program. Meanwhile, our research findings indicate that varenicline has a better short-term smoking cessation effect among smokers who receive free smoking cessation program interventions. As a partial agonist, varenicline has high affinity and selectivity for α4β2 neuronal nicotinic acetylcholine receptors, which is beneficial for increasing the success rate of smoking cessation in smokers who have an active willingness to quit and decide to quit abruptly^26,27^. This drug also has the effect of reducing cigarette consumption for smokers who have not tried to quit smoking and helps to increase the chances of smokers trying to quit smoking^28,29^. In the study of Ebbert et al.^30^, varenicline has a significantly higher smoking cessation efficiency than placebo, which improved the smoking cessation rate in patients during a 6-month follow-up. The mechanism of smoking cessation may be related to its blocking effect on nicotine through partial agonists acting on nicotinic acetylcholine receptors, thereby reducing the body’s addiction to cigarettes^31^. In the network meta-analysis of 20 randomized controlled trials conducted by Guo et al.^32^, the use of varenicline and bupropion in 16702 smokers showed better smoking cessation effects than placebo, and the combination of varenicline and other intervention measures further enhanced smoking cessation effects, similar to our research results. The results of the univariate analysis revealed that married, employed full-time, average monthly cigarette smoking cost <400 RMB, cigarettes smoked per day ≤20, duration of cigarette smoking ≤25 years, pack-year ≤600, and medication with varenicline may be important factors for the success of quitting smoking, which to some extent helps to improve the success rate of smoking cessation. In the study by Sornpaisarn et al.^33^, an analysis was conducted on the factors that influence the success of smoking cessation among Thai social smokers.

### Limitations

This study has some findings on smoking cessation interventions but notable limitations. Most participants were from a single Beijing hospital, risking regional bias. It only tested varenicline and bupropion, overlooking other treatments. Future research should explore more options and combination strategies. Additionally, it only assessed 3-month abstinence rates without long-term follow-up. Since quitting smoking is a prolonged process, extended tracking is needed to evaluate intervention sustainability.

It was found that smoking cessation support factors, including medical advice to quit due to the smoker’s illness, having grandchildren or children, exercise, and good appetite, all contributed to the success rate of smoking cessation; conversely, insomnia, social pressure from smoking, association between smoking and habits/specific activities, and enjoyment of smoking were factors that hinder successful smoking cessation, which is in line with the findings of this study.

## CONCLUSIONS

This study found that the free smoking cessation program has a significant reducing effect on nicotine dependence among smokers, but its impact on smoking cessation confidence, exhaled CO, and smoking cessation effectiveness is comparable to that of general outpatient interventions. In addition, medication with varenicline helps to improve the success of three-month continuous quitting in both general outpatient and free smoking cessation programs. Our findings help to provide a more credible clinical basis for designing smoking cessation guidelines and promote the smoking cessation work of both free smoking cessation programs and general smoking cessation outpatient clinics, providing a theoretical basis for better developing free smoking cessation programs in the future and more ideas for smoking cessation outpatient clinics.

## Data Availability

The data supporting this research are available from the authors on reasonable request.
